# 5'PPP-RNA induced RIG-I activation inhibits drug-resistant avian H5N1 as well as 1918 and 2009 pandemic influenza virus replication

**DOI:** 10.1186/1743-422X-7-102

**Published:** 2010-05-21

**Authors:** Priya Ranjan, Lakshmi Jayashankar, Varough Deyde, Hui Zeng, William G Davis, Melissa B Pearce, John B Bowzard, Mary A Hoelscher, Victoria Jeisy-Scott, Mayim E Wiens, Shivaprakash Gangappa, Larisa Gubareva, Adolfo García-Sastre, Jacqueline M Katz, Terrence M Tumpey, Takashi Fujita, Suryaprakash Sambhara

**Affiliations:** 1 Influenza Division, NCIRD, Centers for Disease Control and Prevention, 1600 Clifton Road, Atlanta, GA 30333, USA; 2 Mount Sinai School of Medicine, One Gustave L Levy Place, New York, NY 10029, USA; 3 Laboratory of Molecular Genetics, Institute for Virus Research, Kyoto University, Kyoto, Japan

## Abstract

**Background:**

Emergence of drug-resistant strains of influenza viruses, including avian H5N1 with pandemic potential, 1918 and 2009 A/H1N1 pandemic viruses to currently used antiviral agents, neuraminidase inhibitors and M2 Ion channel blockers, underscores the importance of developing novel antiviral strategies. Activation of innate immune pathogen sensor Retinoic Acid Inducible Gene-I (RIG-I) has recently been shown to induce antiviral state.

**Results:**

In the present investigation, using real time RT-PCR, immunofluorescence, immunoblot, and plaque assay we show that 5'PPP-containing single stranded RNA (5'PPP-RNA), a ligand for the intracytoplasmic RNA sensor, RIG-I can be used as a prophylactic agent against known drug-resistant avian H5N1 and pandemic influenza viruses. 5'PPP-RNA treatment of human lung epithelial cells inhibited replication of drug-resistant avian H5N1 as well as 1918 and 2009 pandemic influenza viruses in a RIG-I and type 1 interferon dependant manner. Additionally, 5'PPP-RNA treatment also inhibited 2009 H1N1 viral replication *in vivo *in mice.

**Conclusions:**

Our findings suggest that 5'PPP-RNA mediated activation of RIG-I can suppress replication of influenza viruses irrespective of their genetic make-up, pathogenicity, and drug-sensitivity status.

## Background

Annual influenza epidemics caused by influenza A and B viruses result in three to five million cases of severe illness with about 250,000 to 500,000 deaths globally every year. In the United States, complications from influenza infections result in approximately 250,000 hospitalizations and 36,000 deaths in an average year, with majority of the fatalities occurring among the elderly population [1]. Influenza A viruses are further sub typed based on hemagglutinin (HA) and neuraminidase (NA) proteins present on the virion envelope and there are 16 HA and 9 NA types known among influenza A viruses [2,3]. Frequent minor genetic changes, known as antigenic drift and the emergence of influenza A viruses with novel NA and/or HA subtypes, known as antigenic shift result in epidemics and pandemics respectively. In the 20^th ^century, only viruses of the H1, H2 or H3 and N1 or N2 subtypes have caused sustained epidemics in humans. However, other subtypes namely H7, H9, and H5 which primarily cause infections and death among avian species have crossed the species barrier and caused mild to severe or fatal disease in humans [4]. Since 2003, highly pathogenic avian influenza (HPAI) H5N1 viruses have expanded their geographical distribution and are currently endemic in domestic poultry and wild birds in approximately 60 countries on three continents [5]. As of May 61, 2010, 498 human cases in 15 countries with a 60% mortality rate have been reported [6]. Consequently, these viruses have the potential to cause a pandemic, if they acquire the ability for sustained transmission among humans [7,8]. In fact we are in the midst of a pandemic as a result of sustained human-to-human transmission by a novel H1N1 virus containing the gene segments from avian, human, and swine influenza viruses to which people lack immunity [9-11].

Vaccination is the primary strategy for reducing the morbidity and mortality associated with human influenza [12-15]. However, the population at risk such as elderly, pediatric, transplant recipients, and others who are immunocompromised with either primary or secondary immunodeficiency disorders remain vulnerable despite vaccination as in case of avian influenza viral infection and children, adolescents, pregnant women, and those with underlying medical conditions as in case of 2009 H1N1 pandemic influenza virus infection [16]. Therefore, the use of antiviral drugs is a crucial public health countermeasure for preventing and treating influenza, particularly in circumstances of increased incidence influenza infections when there is a vaccine mismatch or shortage, when vaccine usage is limited or non-existent, or when there is no effective vaccine available globally in the market as in the case of H5N1 virus infections. Currently, two classes of antiviral drugs are available to treat influenza infections: the M2 ion-channel blockers amantadine and rimantadine and the NA inhibitors oseltamivir and zanamivir [17-20]. However, the emergence of human seasonal, highly virulent H5N1 influenza viruses as well as 2009 H1N1 pandemic influenza viruses that are resistant to one or both the classes of drugs underscores the need for development of new generation drugs as well as other novel preventive and therapeutic strategies [13,21-28].

The immune system has evolved to recognize and eliminate pathogens. A number of pathogen recognition receptor (PRRs) families are involved in pathogen sensing and can be present in the host as soluble molecules in tissue fluids and serum or as molecules on cell membranes, localized in various cellular compartments, or in the cytosol [29-31]. Recognition of pathogen-associated molecular patterns (PAMPs) by PRRs results in rapid induction of innate immune responses that include production of antiviral cytokines such as the type I interferons (IFN-I) as well as proinflammatory cytokines responsible for impairment of viral replication and induction of adaptive immune responses [32]. The presence of viral RNA or DNA in cytosol is detected by retinoic acid inducible gene-I (RIG-I) and melanoma differentiation-associated gene 5 (MDA-5), DNA-dependent activator of IFN-regulator factors (DAI) or absent in melanoma 2 (AIM2) [33-36]. Several human viruses, including hepatitis C (HCV), vaccinia, Ebola, and influenza have evolved strategies to target and inhibit distinct steps in the early signaling events that lead to IFN-I induction, indicating the importance of IFN-I in the host's antiviral response [37-40]. In case of influenza viruses, we and others have shown that nonstructural protein 1 (NS1) inhibits the function of the RIG-I [41-44]. RIG-I is critical for the induction of an antiviral innate immune response against influenza virus and its C-terminal helicase domain contains the characteristic amino acid signature motif of many RNA binding proteins [45]. The interaction of C-terminal domain with viral RNA either short double stranded RNA or 5'PPP-ssRNA with a panhandle structure facilitates its interaction with IPS-1 (interferon-β promoter stimulator 1) via its N-terminal CARD (caspase-recruitment domain) [42,46-48]. Recent reports suggest various ligands for RIG-I including ssRNA and dsRNA that may require specific sequences or may not require a triphosphate on their 5'-termini [42,46-53]. Despite the various reports that describe ligands and mechanisms of RIG-I mediated antiviral response there is no report that suggests that RIG-I activation can inhibit replication of influenza viruses irrespective of their genetic makeup, pathogenecity and drug-resistant status. In the present study, we investigated whether the evolutionarily conserved antiviral strategies such as the stimulation of RIG-I with 5'PPP-RNA inhibit the replication of these influenza viruses.

## Results

### 5'PPP-RNA inhibits the replication of 1918 pandemic virus as well as both wild-type and drug-resistant H5N1

**Figure 1:**
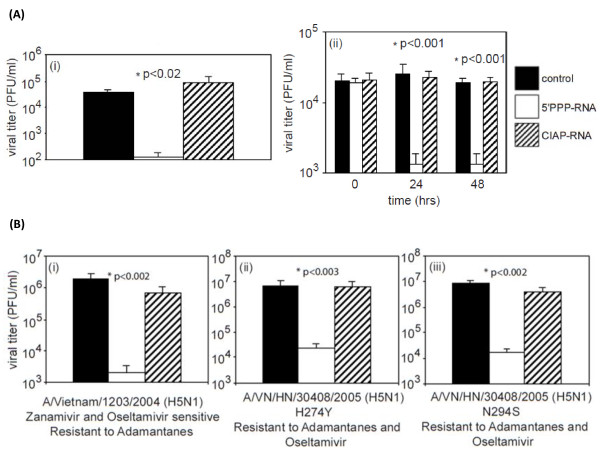
**5'PPP-RNA suprresses the replication 1918 virus as well as wild-type and drug-resistant H5N1 viruses**. (A) A549 (1 × 10^6 ^cells/well) in a 6-well tissue culture plate were mock-transfected (control) or transfected with 2 μg of 5'PPP-RNA or CIAP-RNA using lipofectamine 2000 and 24 hr later were infected with (i) 1918 pandemic virus (0.01 MOI). (ii), Transfected A549 cells were also infected with wild-type H5N1 (0.1 MOI) following 0, 24 and 48 hr of transfection. B. Mock, 5'PPP-RNA or CIAP-RNA transfected A549 cells were infected with drug-resistant H5N1 viruses A/VN/1203/2004 (i), A/VN/30408/2004 H274Y(ii) and A/VN/30408/2004 N294S (iii) 24 hr post transfection. Supernatants collected after 24 hr of infection were assayed for viral titers and results shown are mean ± SD of three independent experiments and are expressed as viral titer (pfu/ml).

The 1918 pandemic resulted in 20-50 million deaths worldwide. To test if the activation of RIG-I with 5'PPP-RNA could suppress the replication of the reconstructed 1918 virus, we treated A549 cells with 5'PPP-RNA or CIAP-RNA for 24 hr, and then infected them with 1918 virus at an MOI of 0.01. Supernatants were collected 24 hr post-infection and assayed for viral titer. Figure 1A(i) indicates that 5'PPP-RNA was able to inhibit 1918 virus replication by ~99% compared with control or CIAP-RNA treated cells. Similar inhibitory effect was observed in experiments where 0.1 MOI was used for infection (data not shown).

Next we evaluated the prophylactic antiviral potential of 5'PPP-RNA against avian influenza H5N1 viruses with pandemic potential. A549 cells were transfected with 5'PPP-RNA or CIAP-RNA for 0, 24 or 48 hr, and infected with A/Vietnam/1203, an H5N1virus. Viral titers were determined 24 hr post-infection. As shown in Figure 1A(ii), A549 cells treated with 5'PPP-RNA showed antiviral effect after 24 hr of transfection. This effect was not seen in control or in CIAP-RNA transfected A549 cells. This inhibitory effect persisted even after 72 hr (data not shown) suggesting a sustained antiviral effect of 5'PPP-RNA.

Stockpiling of the antiviral drug, oseltamivir is one strategy for pandemic preparedness, but the emergence of oseltamivir-resistant H5N1 viruses would seriously impede these efforts. Hence, we also tested the ability of 5'PPP-RNA to inhibit the replication of drug-resistant A/Vietnam/1203/2004 viruses that include zanamivir- and oseltamivir-sensitive and adamantanes-resistant and adamantanes- and oseltamivir-resistant virus variants, A/VN/30408/2005 (H274Y) and A/VN/HN/30408/2005 (N294S). As shown in Figure 1B(i-iii), 5'PPP-RNA transfection in A549 cells significantly reduced the replication of all the three drug-resistant H5N1 viruses. A549 cells treated with CIAP-RNA did not have any impact on viral replication and viral titers were comparable to control. We also used NHBE cells in our studies with H5N1 infection, and similar antiviral effects of 5'PPP-RNA were observed (data not shown). Seasonal drug-resistant H1N1 and H3N2 and their wild-type counter parts were chosen to measure the efficacy of 5'PPP-RNA treatment on viral replication. Influenza viruses of different HA and NA and members of the same subtype have different replication efficiencies. The replication efficiencies of these viruses were well characterized in MDCK cells. As expected, 5'PPP-RNA treatment of A549 cells inhibited the replication of wild-type and drug-resistant human seasonal H1N1, H3N2 and B viruses (Additional file 1, Figure S1). In all cases, activation of RIG-I pathway by 5'PPP-RNA transfection inhibited viral replication by 1.5 to 3 logs. Furthermore, we investigated if the viruses that grew in the presence of 5'PPP-RNA treatment were escape mutants that developed resistance to type I interferon. As shown in Additional file 2, Figure S2, the viruses that grew in the presence of 5'PPP-RNA are still susceptible to 5'PPP-RNA treatment when tested subsequently.

### 5'PPP-RNA inhibits 2009 pandemic H1N1 viruses both *in vitro *and *in vivo*

**Figure 2:**
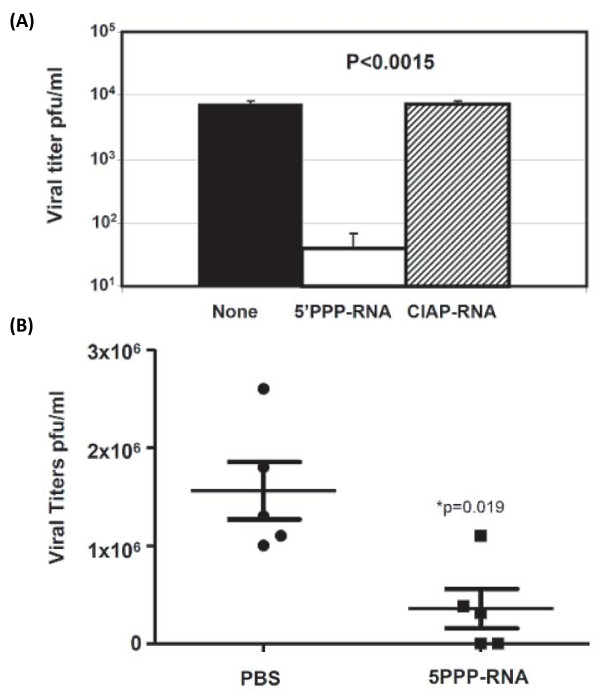
**5'PPP-RNA inhibits replication of 2009 pandemic influenza virus**. (A). A459 cells (1 × 10^6 ^cells/well) in a 6-well tissue culture plate were mock-transfected or transfected with 2 μg of 5'PPP-RNA or CIAP-RNA for 24 hr and then infected with A/California/08/09 at an MOI of 1.0. Supernatants collected 24 hr post-infection were assayed for viral titers as indicated in material and methods. Results shown are mean ± SD from three independent experiments and are expressed as viral titer (pfu/ml) (B). 5'PPP-RNA also reduced replication of A/Mexico/4482/09 virus in Balb/c mice. 8 week old female Balb/c mice received 100 μg of 5'PPP-RNA or PBS alone intravenously for 4 days and the animals were infected with 1000 MID_50 _of 2009 pandemic virus (A/Mexico/4482/09) on day 1. The lungs were collected on day 4 to determine viral titers *P < 0.05 (n = 5).

Global spread of novel 2009 A/H1N1 influenza viruses containing a constellation of genes from avian, human, and swine in relatively short period of time is causing severe disease and fatal outcomes in high-risk populations. It was clear that vaccine was not available and in sufficient quantities for use in the first wave of a pandemic leading to the reliance on the prophylactic and therapeutic use of effective antiviral drugs. However, emergence of oseltamivir-resistant novel 2009 A/H1N1 virus strains underscores the fragility of the public health strategy to control pandemic [27]. Hence, we tested the ability of 5'PPP-RNA to inhibit novel 2009 A/H1N1 virus. As shown in Figure 2A, 5'PPP-RNA transfected A549 cells significantly inhibited A/California/08/09 replication. Control or CIAP-RNA did not show this suppression effect. To investigate if *in vivo *administration of 5'PPP-RNA will reduce viral titers, we delivered 5'PPP-RNA or PBS formulated in *in vivo*jet-PEI daily for four days and infected mice on day 1 with A/Mexico/4482/09. Daily administration of 5'PPP-RNA alone had no side effects on body weight gain and activity (data not shown). On day 4 post-challenge, lungs from all animals were collected to determine viral titers. Control group had significantly higher viral load in lungs when compared to those that received 5'PPP-RNA (Figure 2B). The viral titers from the 5'PPP-RNA group ranged from 40 through10^6 ^pfu/ml. Two animals showed viral titers of 40 and 50 and others showed 3 × 10^5 ^to 1 × 10^6 ^pfu/ml. These data suggest that 5'PPP-RNA treatment inhibited viral replication.

### 5'PPP-RNA induced upregulation of IFNβ and RIG-I

**Figure 3:**
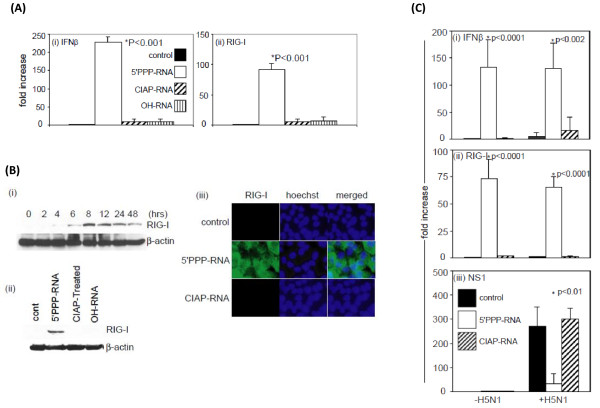
**5'PPP-RNA induced RIG-I and IFNβ expression in A549 cells**. (A) A549 (1 × 10^6 ^cells/well) in a 6-well tissue culture plate were mock-transfected (control) or transfected with 2 μg of 5'PPP-RNA, CIAP-RNA or chemically synthesized OH-RNA using lipofectamine 2000. After 24 hr of treatment, (i) IFNβ and (ii) RIG-I mRNA expression was analyzed by real-time RT-PCR. All data were normalized to β-actin, a house keeping gene and expressed as fold increase. Data shown represent the mean ± SD of three independent experiments. (B), (i) A549 cells were transfected with 2 μg of 5'PPP-RNA for the indicated times, and RIG-I expression was analyzed by immunoblot. (ii) A549 cells were mock-transfected or transfected with 2 μg of 5'PPP-RNA, CIAP-RNA or chemically synthesized OH-RNA for 24 hr. RIG-I expression was analyzed by immunoblot. (iii) A549 cells grown on cover-slips were mock-transfected or transfected with 2 μg of 5'PPP-RNA or CIAP-RNA for 24 hr. Cells were then paraformaldehyde fixed and immunostained with anti-RIG-I antibodies. Alexa fluor 594 goat anti-rabbit IgG Antibody (red fluorescence) or Alexa 488 (green fluorescence) were used as secondary antibodies. Nuclei were stained with Hoechst (blue fluorescence). (C) RNA was isolated from A549 cells mock transfected or trasfected with 5'PPP-RNA or CIAP-RNA for 24 hr and then infected with wild-type H5N1 virus for 24 hr (as described in Figure legend 1). Real-time RT-PCR was performed to analyze the expression of (i) IFNβ, (ii) RIG-I and (iii) NS1. All data were normalized to β-actin, a house keeping gene and expressed as fold increase. Data shown represent the mean ± SD of three independent experiments.

Antiviral effect in response to 5'PPP-RNA has been shown to be associated with induced RIG-I expression and subsequent type I interferon production. Therefore, we investigated the ability of *in vitro *transcribed 5'PPP-RNA to induce IFNβ and RIG-I in A549 cells in our experimental model by real-time RT-PCR. As shown in Figure 3A(i&ii), transfection of A549 cells with 5'PPP-RNA resulted in an approximately 200-fold increase in IFNβ mRNA and ~80-fold increase in RIG-I mRNA expression level in 24 hr. No induction was observed for RIG-I or IFNβ level in mock-transfected or A549 cells transfected with CIAP-RNA or with -OH-RNA. Time-kinetics studies of RIG-I protein expression as shown in Figure 3B(i) suggest that 5'PPP-RNA mediated RIG-I induction was detectable by 6 hr, peaked at 8 hr, and was present even after 48 hr although we observed a decline after 72 hr of treatment (data not shown). Similar kinetics were observed for IFNβ expression by real-time RT-PCR (data not shown). Figure 3B(ii) demonstrates the specific effect of 5'PPP-RNA on RIG-I expression at the protein level in A549 cells that is consistent with real time RT-PCR data [Figure 3A(ii)]. A similar pattern of RIG-I induction by 5'PPP-RNA was observed in NHBE cells (data not shown). We also investigated 5'PPP-RNA induced RIG-I expression in A549 cells by immunostaining [Figure 3B(iii)]. A549 cells treated with 5'PPP-RNA showed increased cytosolic expression of RIG-I. Mock or CIAP-RNA transfected cells did not show detectable levels RIG-I. These experiments were also carried out in NHBE cells and similar effects of 5'PPP-RNA was observed (data not shown).

Activation of the innate immune system is critical for the induction and maintenance of host antiviral defenses. However, viruses also have evolved mechanism(s) that circumvent host immune responses. Several studies, including our own have shown independently that non-structural protein (NS1) of influenza A viruses inhibits RIG-I function. We, therefore, investigated the expression of IFNβ and RIG-I as well as NS1 by real-time RT-PCR in 5'PPP-RNA or CIAP-RNA transfected A549 cells that were either uninfected or infected with H5N1 virus at an MOI of 0.1 for 24 hr following transfection. As shown in Figure 3C(i&ii), 5'PPP-RNA induced IFNβ and RIG-I induction remained unchanged in H5N1 infected A549 cells. H5N1 virus infection induced the expression of NS1 by 250-300-fold over uninfected A549 cells [Figure 3(iii)] and did not induce IFNβ and RIG-I and prior treatment with 5'PPP-RNA significantly inhibited viral NS1 expression. Similar effects were observed for 1918 infection in A549 cells (data not shown).

### Involvement of RIG-I and type 1 interferon in 5'PPP-RNA induced antiviral effect

**Figure 4:**
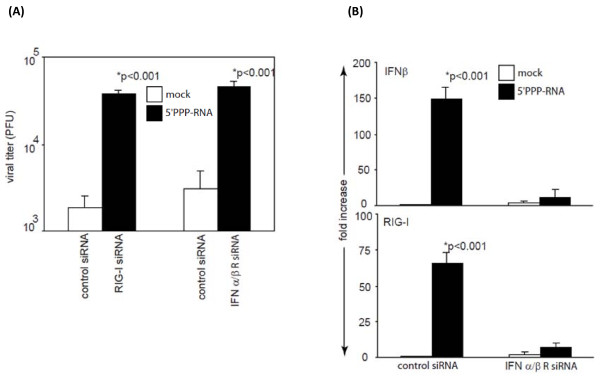
**Indispensible role of type I interferon in RIG-I-dependent 5'PPP-RNA-induced antiviral effects**. (A). A549 cells (1 × 10^6^/well) in a 6-well plate were transfected with control siRNA or siRNA against RIG-I or IFN αβ receptors using DharmaFect 1 transfection reagent as per manufacturer instructions (Dharmacon, Lafayette, CO, USA). After 24 hr, cells were transfected with 2 μg of 5'PPP-RNA. A549 cells were then infected with A/New York/02/2001, a H1N1 virus at 0.1 MOI. Supernatants collected after 24 hr were analyzed for virus growth by plaque assay using MDCK cells. (B) A549 cells from experiment (A) were also analyzed for IFNβ and RIG-I expression by quantitative RT-PCR as described elsewhere. Data shown represent the mean ± SD of three independent experiments.

RIG-I activation has been reported to stimulate type 1 interferon expression that induces an antiviral state. To study the direct role of 5'PPP-RNA induced RIG-I and type 1 interferon induction we knockdown RIG-I or IFNαβ-receptor using gene specific siRNA. As shown in Figure 4A, selective knockdown of RIG-I in A549 cells failed to induce antiviral effects when treated with 5'PPP-RNA and infected with A/New York/02/2001. This confirms that 5'PPP-RNA mediated antiviral effects require RIG-I. 5'PPP-RNA treatment also resulted in induced IFNβ expression. To understand the correlation between increased IFNβ level and antiviral effect, we knocked down IFN-α/β-receptors in A549 cells using siRNA, and treated the cells with 5'PPP-RNA followed by infection with A/New York/02/2001. As shown in Figure 4A, knocking down IFNα/β-receptors significantly abrogated the antiviral effect of 5'PPP-RNA as compared to control siRNA knockdown. Furthermore, 5'PPP-RNA treatment of A549 cells with IFN-α/β-receptors knockdown failed to induce the expression of RIG-I and IFNβ as assessed by quantitative RT-PCR (Figure 4B). We also measured IFNβ, RIG-I and NS1 expression by qRTPCR and western blot analyses for all the viruses used in our studies. H5N1 and H1N1 viruses behaved the same way RIG-I or αβ IFN-receptor gene knock-down experiments (data not shown) as A/New York/02/2001 virus. These data clearly indicate that type 1 interferon is not only required for antiviral effect but also for upregulating RIG-I expression by autocrine and paracrine manner.

### *In vitro *therapeutic potential of 5'PPP-RNA

**Figure 5:**
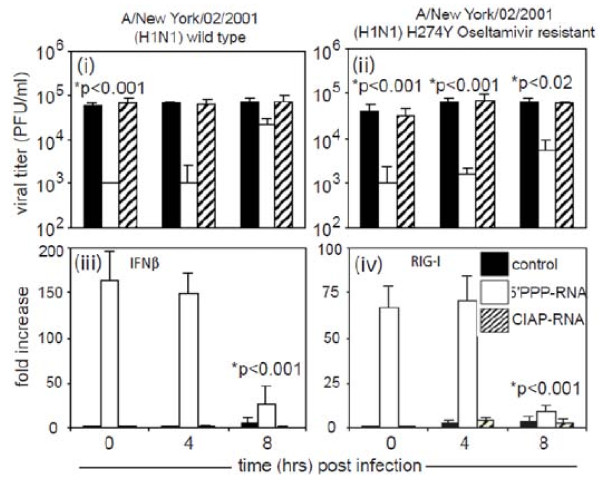
**Therapeutic potential of 5'PPP-RNA**. A459 cells (1 × 10^6 ^cells/well) in a 6-well tissue culture plate were infected with (i) wild-type A/New York/02/2001 and its (ii) H274Y oseltamivir-resistant A/New York/02/2001 variant for the 0 hr, 4 hr or 8 hr before treatment with 5'PPP-RNA or CIAP-RNA. Supernatants collected after 24 hr of infection were assayed for viral titers as indicated in material and methods. Cells were harvested to assay IFNβ (iii) and RIG-I (iv) mRNA level by Real time RT-PCR. Results shown are mean ± SD from three independent experiments and are expressed as viral titer (pfu/ml) or fold increase over controls.

We also investigated the therapeutic potential of 5'PPP-RNA in A549 cells infected with seasonal influenza virus A/New York/02/2001 and its oseltamivir-resistant H274Y mutant. As shown in Figure 5, A549 cells were first infected with/New York/02/2001 (i) or its oseltamivir-resistant counterpart H274Y (ii). 5'PPP-RNA trasfection was carried out post 0 hr, 4 hr and 8 hr infection. Data suggest that 5'PPP-RNA treatment inhibited both wild-type and drug-resistant virus replication when delivered up to 4 hr post infection (p < 0.05), whereas a weaker inhibitory effect was observed after 8 hr post-infection. We also investigated the IFNβ and RIG-I expression in these experiments. As shown in Figure 5(iii&vi), 5'PPP-RNA transfection post 8 hr A/New York/02/2001 infection induced significantly low level of IFNβ (iii) and RIG-I (iv) as compared to those of 0 hr or 4 hr transfection. Similar results were observed for oseltamivir-resistant H274Y mutant (data not shown). A time-kinetics studies of NS1 expression following virus infection suggest that expression of NS1 reaches optimal by 8 hr of infection (data not shown). This correlates with diminished antiviral effect of 5'PPP-RNA and down-regulation of type I interferon and RIG-I level at 8 hr of infection suggesting NS1 interferes with RIG-I activation.

## Discussion

Three influenza pandemics occurred during the 20th century, with varying degrees of severity; outcomes ranged from the high levels of illness and death observed during the 1918 Spanish flu pandemic (estimates of deaths range from 20 to 100 million) to the much lower levels observed during the pandemics of 1957 and 1968 (approximately one million deaths each) [54,55]. Furthermore, the spread of highly pathogenic avian influenza viruses since 2004 has also intensified concern over the emergence of novel strains of influenza with pandemic potential as exemplified by the current pandemic caused by a triple reassortant virus [9]. Since the vaccine was not available for use in the first wave of a pandemic, Oseltamivir, a neuraminidase inhibitor became the first choice for prophylactic and therapeutic intervention. The ongoing emergence of seasonal avian H5N1 as well as novel H1N1 pandemic influenza viruses that are resistant to one or other class of antiviral drugs underscores the fragility of the public health strategy to control seasonal influenza infections [28,56,57]. To overcome these challenges and improve preparedness against infections with oseltamivir-resistant viruses, developing novel antiviral prophylactic as well as therapeutic approaches that offer a broad spectrum approach and are independent of the genetic makeup of influenza viruses is absolutely critical.

In the present study, we explored the role of *in vitro *transcribed 5'PPP-RNA, a ligand for cytoplasmic RNA sensor, RIG-I, in antiviral innate immune response against a panel of influenza viruses including drug-resistant, potential pandemic as well as pandemic strains in human lung epithelial cells. The broad-spectrum prophylactic potential of 5'PPP-RNA against influenza viruses is clearly evident from our studies. 5'PPP-RNA significantly inhibited *in vitro *growth of not only wild-type drug-sensitive and resistant H5N1 strains with comparable efficacy but also highly pathogenic 1918 pandemic strain. We also included 2009 pandemic H1N1 viruses in our studies to see the effect of 5'PPP-RNA both *in vitro *and *in vivo*. Data shown Figure 2A clearly indicate that *in vitro *suppression level of A/California/08/09 was similar to that of other viruses used in this study. Furthermore, 5'PPP-RNA also inhibited A/California/08/09 viral replication *in vivo *in Balb/c mice. These data suggest that irrespective of type or subtypes, drug-susceptibility status, or *in vivo *virulence, the replication of influenza viruses was inhibited by 5'PPP-RNA. The dose and frequency of administration of 5'PPP-RNA may influence the extent of inhibition of viral replication.

Treatment with 5'PPP-RNA not only induced the expression of IFN-I but also upregulated RIG-I expression. 5'PPP-RNA induced expression of IFN-I and RIG-I was not affected by subsequent infection with H5N1 (Figure 3C) or 1918 pandemic influenza viruses (data not shown). Induction of these molecular regulators of innate immune pathway may be involved in the sustained action of the 5'PPP-RNA-induced antiviral effect.

Viruses have acquired mechanism(s) to escape host immune surveillance by inhibiting IFN induction pathways. The NS1 protein of Influenza A virus inhibits host antiviral defenses by direct interaction with RIG-I [41-44]. 5'PPP-RNA treated A549 cells infected with H5N1 virus showed significant reduction of NS1 mRNA expression as compared to controls, thereby limiting the ability of the virus to interfere with the host innate immune system. RIG-I-mediated antiviral effect by 5'PPP-RNA lasted for 48 hr which also correlates with sustained induction of IFN-I that lasted more than 48 hr as revealed by time-kinetics studies (data not shown). To test the role of RIG-I, we knocked down RIG-I expression using siRNA, which abrogated 5'PPP-RNA ability to induce IFNβ and to inhibit virus replication. Since, there is a significant increase in IFNβ in addition to RIG-I in 5'PPP-RNA treated lung epithelial cells, we investigated whether this effect is due to the action of IFN-I induced by initial interaction of 5'PPP-RNA with RIG-I. We abrogated the expression of IFNα/β-receptors of A549 cells using siRNA and infected them subsequently with A/New York/02/2001 virus. By knocking down the expression of IFNα/β receptors, we were unable to detect significant level of IFNβ and RIG-I induction following 5'PPP-RNA treatment and inhibitory effect on virus growth (Figure 4A&4B). These findings suggest the direct involvement of RIG-I-induced type I IFN in the 5'PPP-RNA mediated antiviral effect. Our data support the previous findings that IFN can act in an autocrine or paracrine manner to induce its own expression as well as other antiviral modulators to generate an antiviral state [58]. Though type I interferon seems to be key player in antiviral action, systemic or local delivery of IFNβ may be toxic and may result in uncontrolled inflammation [59]. Activation induced type I interferon is self-regulated by feedback mechanisms and prevents side effects.

To evaluate the therapeutic potential of 5'PPP-RNA, we treated pre-infected (with wild-type and drug-resistant A/New York/02/2001) A549 cells with 5'PPP-RNA at different time-points. We observed relatively weak antiviral effect of 5'PPP-RNA, when A549 cells were transfected with 5'PPP-RNA post 8 hr infection. Interestingly, at this time point we also observed significantly low RIG-I and IFNβ expression (Figure 5). These data again suggest the critical role of RIG-I and type I IFN expression which may have been suppressed due to NS1 expression at 8 hr post-infection.

## Conclusions

The findings described herein demonstrate that understanding host innate immune functions at the molecular level is a most promising strategy to identify targets, such as RIG-I for developing newer classes of drugs. These novel approaches that boost host antiviral innate immune defenses are broad spectrum in nature, rather than virus-specific as they inhibited replication of wild-type and drug-resistant strains of seasonal and avian viruses as well as pandemic influenza viruses. Since these approaches are based on stimulating host antiviral defenses, the possibility of viruses developing resistance to this approach is remote as these defenses are evolutionarily conserved.

## Methods

### *In vitro *synthesis of 5'PPP-RNA

5'PPP-RNAs were synthesized using T7 RNA polymerase. Using annealed complimentary oligo nucleotides, a DNA template was constructed that contains a T7 RNA polymerase promoter followed by the sequence of interest (AGCUUAACCUGUCCUUCAA) to be transcribed [60,61]. Twenty pmol of the DNA template were incubated with 25U T7 RNA polymerase, 40U RNase inhibitor in a buffer containing 40 mM Tris-Hcl (pH 8.0), 10 mM DTT, 2 mM spermidine-HCl, 20 mM MgCl_2 _and NTPs. DNA template was digested with DNase I and RNA was purified using phenol:chloroform extraction and ethanol precipitation. Size, integrity and single strandness of RNA was analyzed by RNase A digestion followed by gel electrophoresis. Calf intestine alkaline phosphatase (CIAP) treatment was performed to remove tri-phosphate groups from *in vitro *synthesized RNA. Briefly, 100 μg of *in vitro *transcribed RNA was treated with 150U of CIAP for 3 hr at 37°C in a buffer containing 50 mM Tris-HCl (pH 8.0), 0.1 mM EDTA and 50U of RNase inhibitor. RNA was purified as described above. Chemically synthesized RNA with same sequence and length that contains an OH-group at its 5'-end was purchased from Dharmacon (Lafayette, CO, USA).

T7 polymerase driven *in vitro *transcribed 5'-PPP-RNA has been shown to contain dsRNA as well that activates RIG-I [46,48]. To investigate if our *in vitro *transcribe 5'PPP-RNA is single stranded RNA (ssRNA) and/or double stranded RNA,(dsRNA) we used RNase A treatment under the conditions that degrades ssRNA, but not dsRNA. This confirmed that *in vitro *transcribed RNA used in our study was RNA only (Additional file 3, Figure S3).

### Cell lines

A549 cells were grown in DMEM (Life Technologies, Grand Island, NY) supplemented with 10% fetal bovine serum (FBS), 100? U/ml penicillin and 100 μg/ml streptomycin. Normal human bronchial epithelial (NHBE) cells (Base, Lonza, Switzerland) were maintained under the conditions and media specified by the supplier.

### Influenza viruses

Seasonal viruses used in this study include the laboratory H1N1 strain A/Puerto Rico/8/34, wild-type H1N1 (A/Texas/36/91 and its oseltamivir-resistant variant H274Y; wild-type A/New York/02/2001 and its H274Y oseltamivir-resistant variant), H3N2 (adamantane-resistant A/Wisconsin/06/94, A30V and A/Wisconsin/12/90, L26F), and B viruses (wild-type B/Memphis/20/96 and its R152K mutant resistant to NA inhibitors zanamivir and oseltamivir), HPAI H5N1 viruses (zanamivir- and oseltamivir-sensitive and adamantane-resistant A/Vietnam/1203/2004 and, adamantane- and oseltamivir-resistant virus variants A/Vietnam/30408/2005 (H274Y), and A/Vietnam/HN30408/2005 (N294S),, 2009 pandemic H1N1 viruses, A/California/08/09 and A/Mexico/4482/09, and the reconstructed 1918 Spanish influenza pandemic virus (H1N1) generated by plasmid-based reverse genetics [62]. All human and avian wild-type and drug-resistant viruses were obtained from the influenza division CDC repository.

### 5'PPP-RNA treatment and influenza viral infections

Cells were transfected with 2 μg of *in vitro *transcribed 5'PPP-RNA, chemically synthesized RNA or CIAP-treated RNA using Lipofectamine 2000. This dose was determined by dose-kinetics studies using 1, 2, 4 and 6 μg of RNA. After 24 hr, cells were infected with the different viruses used in this study in a 6-well plate. Unless specified, infection of cells was performed at a multiplicity of infection (MOI) of 0.1 with or without trypsin supplement. Each treatment was carried out in duplicate cultures. After 24 hr, cells were harvested for RNA and protein analysis and cell-culture supernatants were collected and stored at -80°C for determination of viral titer by plaque assay as described previously using MDCK cells[41]. This time-point was determined by kinetics studies using PR8. To study the therapeutic potential of 5'PPP-RNA cells were infected with viruses first for 2, 4 or 8 hr followed by transfection with 5'PPP-RNA. Three independent experiments were performed at different times with each treatment carried out in duplicate cultures.

### Real Time RT-PCR

Total RNA was isolated from cells using the RNAeasy kit (Qiagen, Valencia, CA, USA) and real time RT-PCR was conducted using a Stratagene Q3005 PCR machine for mRNA expression of RIG-I, IFNβ, NS1, and β-actin. For each sample, 2 μg of RNA was reverse transcribed using Superscript II Reverse Transcriptase (Invitrogen, Carlsbad, CA, USA) according to the manufacturer's directions. Parallel reactions without reverse transcriptase were included as negative controls. Reverse transcription reactions (1/50^th ^of each reaction) were analyzed in using syber green Q-PCR reagents (Stratagene, La Jolla, CA, USA). PCR condition was kept as 94°C for 15 s, annealing at 56°C for 30 s, and extension at 72°C for 30 s for a total of 45 cycles. The threshold cycle number for cDNA was normalized to that of β-actin mRNA, and the resulting value was converted to a linear scale. Data from three independent experiments were taken account for analysis. All data points fell into a normal distribution and there were no outliers.

Primer sets used for these studies are as follows:

IFNβ: forward *5'- TGG GAG GCT TGA ATA CTG CCT CAA -3'*

reverse 5'- TCT CAT AGA TGG TCA ATG CGG CGT -3'

RIG-I: forward *5'- AAA CCA GAG GCA GAG GAA GAG CAA -3'*

reverse 5'- TCG TCC CAT GTC TGA AGG CGT AAA -3'

NS1: forward *5'-AGA AAG TGG CAG GCC CTC TTT GTA-3'*

reverse 5'-TGT CCT GGA AGA GAA GGC AAT GGT -3'

β-actin: forward *5'- ACC AAC TGG GAC GAC ATG GAG AAA -3'*

reverse 5'- TAG CAC AGC CTG GAT AGC AAC GTA -3'

### Immunoblotting

Cells were washed with chilled PBS and then lysed in 100 μl of ice-cold lysis buffer (50 mM Tris-Cl, pH 8.0, 150 mM NaCl, 10% v/v glycerol, 1% v/v Triton X-100, 2 mM EDTA, 1 mM PMSF, 20 μM leupeptin containing aprotinin 0.15 μg/ml) for 20 minutes at 4°C. The protein content of different samples was determined using a protein assay reagent (BioRad, Inc., CA, USA). Equal quantities of solubilized protein were resolved by 10% SDS-PAGE, blotted to nitrocellulose membrane and probed with the indicated primary antibodies. Anti-RIG-I was purchased from Axxora, LLC (San Diego, CA, USA). Anti-β-actin antibody was purchased from Sigma, St. Louis, MO, USA. Antibody signals were detected by chemiluminescence using secondary antibodies conjugated to horseradish peroxidase and an ECL detection kit (Amersham Biosciences, Inc., NJ, USA).

### Fluorescence microscopy

A549 cells grown on cover-slips were mock-transfected or transfected with 2 μg of 5'PPP-RNA or CIAP-RNA for 24 hr. Cell monolayers were then washed with cold PBS and fixed with 2% paraformaldehyde for 15 min at room temperature. Cells were permeablized with 0.05% saponin in PBS for 30 min at room temperature, and then blocked with blocking buffer (1% BSA, 5% normal goat serum and 0.05% saponin in PBS) at room temperature. Cells were incubated with primary antibodies (1 μg/ml) at 4°C overnight, and were washed 3 times with wash buffer (0.05% saponin in PBS). Cell monolayers were further incubated with secondary antibodies (Alexa 594 or 488) in blocking buffer for 1 hr at room temperature followed by three washes with buffer. To stain nuclei, cells were incubated with Hoechst dye (5 μM in PBS containing 0.05% saponin). Cells were further washed (3 times) with PBS, and were mounted on slides using Prolong antifade mounting media (Invitrogen, Carlsbad, CA, USA) and were observed under a Zeiss LSM 510 fluorescence microscope.

### *In vivo *studies

Female Balb/c mice 6-12 weeks old purchased from Jackson Laboratories were intravenously injected 100 μg of 5'PPP-RNA, capped 5'-RNA or PBS complexed with *in vivo*-jetPEI according to manufacturer's protocol (Polyplus-transfection Inc, San Diego, CA USA) in a volume of 200 μl for 4 days. On day 1, mice were challenged intranasaly with 1000 MID_50 _of 2009 pandemic virus (A/Mexico/4482/09). A separate group of animals that received only 5'PPP-RNA complexed with *in vivo*-jetPEI without viral challenge were observed to determine changes in body weight and activity. Lungs from control and treated challenged animals were collected on day 4 to determine viral titers using MDCK cells as described earlier.

### Statistical Methods

To determine the statistical significance among the 5'PPP-RNA or CIAP-RNA treated and untreated groups, we used Analysis of variance and a value of *P *< 0.05 was considered significant. All data points were included in the analysis and there were no outliers.

## Competing interests

The authors declare that they have no competing interests.

## Authors' contributions

PR participated in the planning and execution of the experiments, analyses of the results, and manuscript writing. LJ, VD, and MAH performed studies with human and avian viruses. VJ, WGD, JBB, MEW and MBP assisted in making 5'PPP-RNA and tested mRNA samples for the expression of various host innate immune response genes with cytokine arrays. SG provided assistance in assessing the functionality of cytokines and chemokines. HZ and TMT performed studies with the 1918 pandemic virus. LG, JMK, AG, and TF provided advice in the planning and execution of the studies. SS participated in the planning, coordination, supervision, and execution of the experiments that led to the present manuscript. All the authors saw and approved the final version of the manuscript.

## Supplementary Material

Additional file 1 **Figure S1**. 5'PPP-RNA inhibits replication of drug-resistant human viruses. A459 cells (1 × 10^6 ^cells/well) in a 6-well tissue culture plate were mock-transfected or transfected with 2 μg of 5'PPP-RNA or CIAP-RNA for 24 hr and then infected with (a&b) wild-type and drug-resistant human H1N1 viruses; (b&c) drug-resistant H3N2 viruses and (d&e) wild-type and drug-resistant B viruses. Supernatants collected were assayed for viral titers as indicated in material and methods. Results shown are mean ± SD from three independent experiments and are expressed as viral titer (pfu/ml). Click here for file

Additional file 2 **Figure S2. Testing viral colonies if they are interferon escape mutant**. A/NewYork/02/2001 colonies from 5'PPP-RNA treated A549 cells grown on MDCK cell were isolated and viral stocks were made by growing them in MDCK cells. Subsequently, these viruses were tested for 5'PPP-RNA sensitivity in A549 cells transfected 24hr earlier with either CIAP-RNA or 5'PPP-RNA. Culture supernatants were collected 24hr post-infection to determine viral titers in MDCK cells as described in materials and methods. Click here for file

Additional file 3 **Figure S3. Analysis of size, integrity and single or double-strandness of RNA**. *In vitro *transcribed RNA (1 μg) generated by T7 polymerase was digested with 0.1 μg/ml RNase A (Ambion) or DNase I (10U/ml) (Ambion) at 37C for 1 hr, separated on agarose gel, and visualized by ethidium bromide staining. Click here for file
