# Supplementary figures and images for: 5'PPP-RNA induced RIG-I activation inhibits drug-resistant avian H5N1 as well as 1918 and 2009 pandemic influenza virus replication
Source: Virol J. 2010 May 21;7:102. doi: 10.1186/1743-422X-7-102 (PMC2891689; doi:10.1186/1743-422X-7-102)

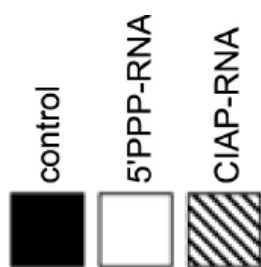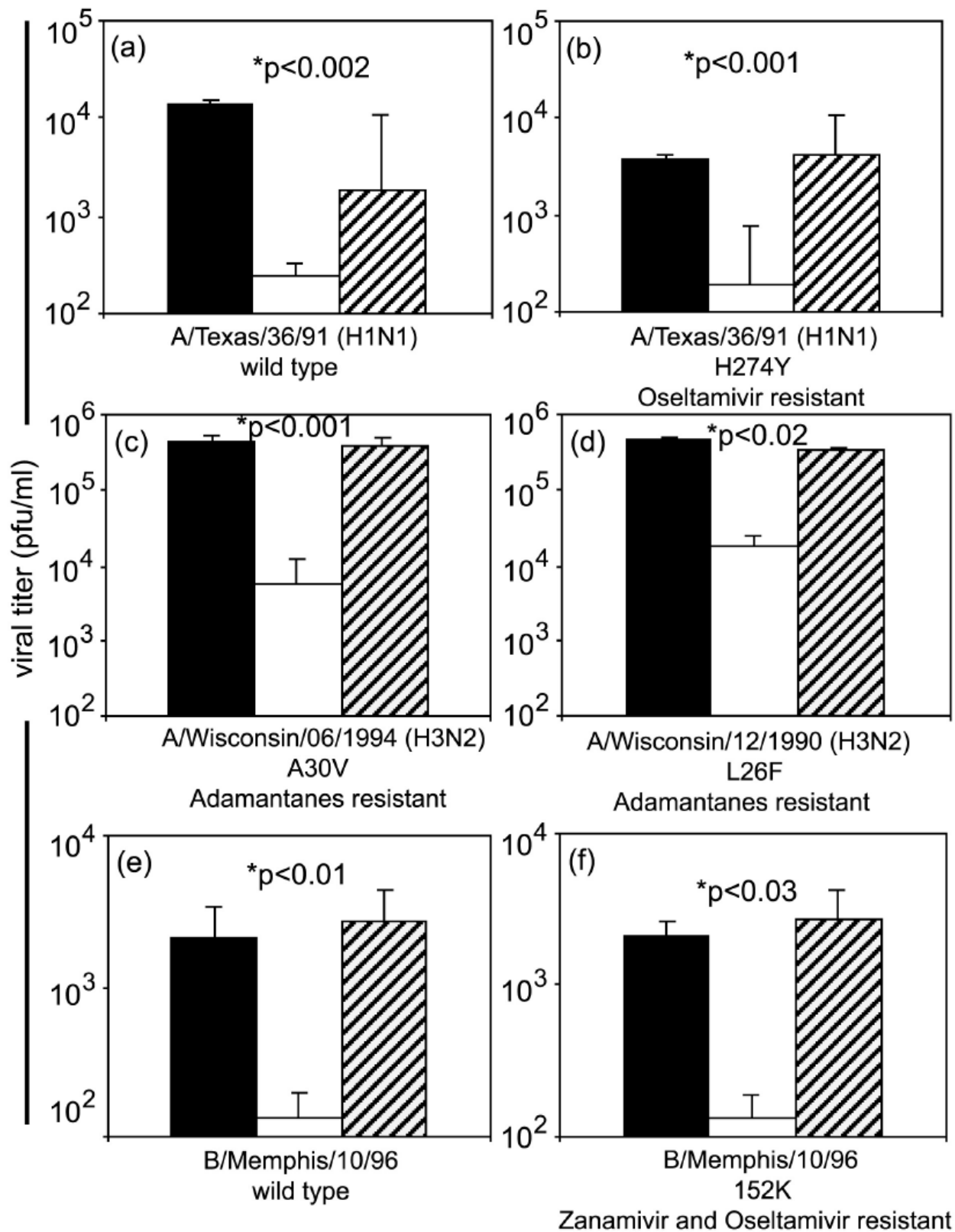

Supplement: Additional file 1 — Figure S1. 5'PPP-RNA inhibits replication of drug-resistant human viruses. A459 cells (1 × 10^6^ cells/well) in a 6-well tissue culture plate were mock-transfected or transfected with 2 μg of 5'PPP-RNA or CIAP-RNA for 24 hr and then infected with (a&b) wild-type and drug-resistant human H1N1 viruses; (b&c) drug-resistant H3N2 viruses and (d&e) wild-type and drug-resistant B viruses. Supernatants collected were assayed for viral titers as indicated in material and methods. Results shown are mean ± SD from three independent experiments and are expressed as viral titer (pfu/ml). [file 1743-422X-7-102-S1.PDF]

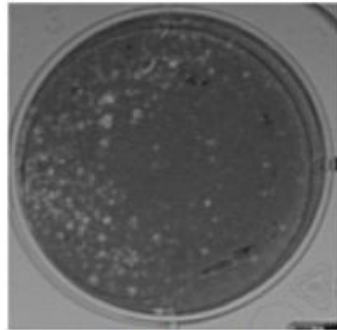

CIAP-RNA

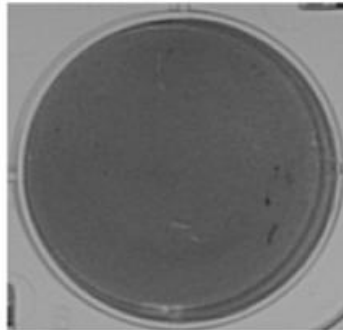

5'PPP-RNA

Supplement: Additional file 2 — Figure S2. Testing viral colonies if they are interferon escape mutant. A/NewYork/02/2001 colonies from 5'PPP-RNA treated A549 cells grown on MDCK cell were isolated and viral stocks were made by growing them in MDCK cells. Subsequently, these viruses were tested for 5'PPP-RNA sensitivity in A549 cells transfected 24hr earlier with either CIAP-RNA or 5'PPP-RNA. Culture supernatants were collected 24hr post-infection to determine viral titers in MDCK cells as described in materials and methods. [file 1743-422X-7-102-S2.PDF]

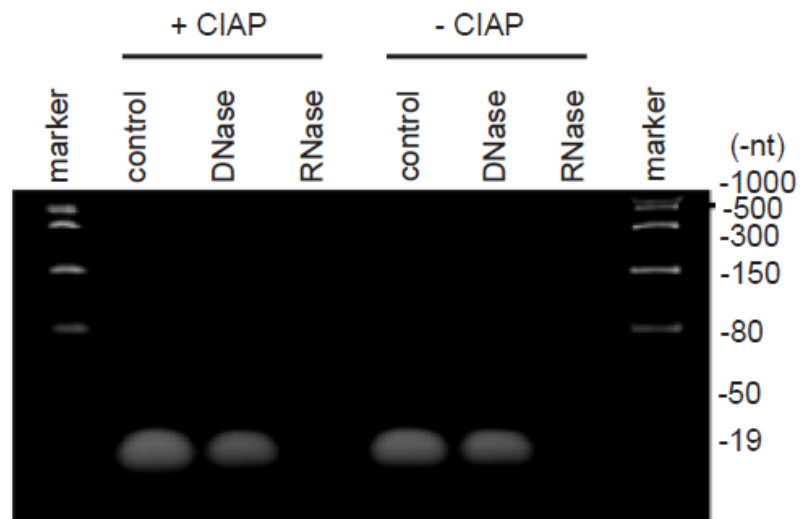

Supplement: Additional file 3 — Figure S3. Analysis of size, integrity and single or double-strandness of RNA. In vitro transcribed RNA (1 μg) generated by T7 polymerase was digested with 0.1 μg/ml RNase A (Ambion) or DNase I (10U/ml) (Ambion) at 37C for 1 hr, separated on agarose gel, and visualized by ethidium bromide staining. [file 1743-422X-7-102-S3.PDF]
